# LC-ESI/MS-Phytochemical Profiling with Antioxidant, Antibacterial, Antifungal, Antiviral and In Silico Pharmacological Properties of Algerian *Asphodelus tenuifolius* (Cav.) Organic Extracts

**DOI:** 10.3390/antiox10040628

**Published:** 2021-04-20

**Authors:** Ayoub Khalfaoui, Emira Noumi, Soumia Belaabed, Kaïss Aouadi, Bouslama Lamjed, Mohd Adnan, Andrea Defant, Adel Kadri, Mejdi Snoussi, Mushtaq Ahmad Khan, Ines Mancini

**Affiliations:** 1 Research Unit, Development of Natural Resources, Bioactive Molecules, Physicochemical and Biological Analysis (VARENBIOMOL), Department of Chemistry, University Mentouri Constantine, Route Ain ElBey, Constantine 25000, Algeria; khalfaoui.ayoub@umc.edu.dz (A.K.); soumia.belaabed@umc.edu.dz (S.B.); 2 Department of Biology, College of Science, Hail University, P.O. Box 2440, Ha’il 81451, Saudi Arabia; emira_noumi@yahoo.fr (E.N.); drmohdadnan@gmail.com (M.A.); 3 Laboratory of Bioressources, Integrative Biology & Recovery, High Institute of Biotechnology-University of Monastir, Monastir 5000, Tunisia; 4 Department of Chemistry, College of Science, Qassim University, Buraidah 51452, Saudi Arabia; kaiss_aouadi@hotmail.com; 5 Laboratory of Hetrocyclic Chemistry, Natural Products and Reactivity, Avenue of the Environment, Faculty of Science of Monastir, University of Monastir, Monastir 5019, Tunisia; 6 Laboratory of Bioactive Substances, Center of Biotechnology of BorjCedria, University of Tunis El Manar, Tunis 1068, Tunisia; lamjed.bouslama@gmail.com; 7 Laboratorio di Chimica Bioorganica, Dipartimento di Fisica, Universita di Trento, I-38123 Povo, 38123 Trento, Italy; andrea.defant@unitn.it (A.D.); ines.mancini@unitn.it (I.M.); 8 Faculty of Science and Arts in Baljurashi, Albaha University, P.O. Box 1988, Albaha 65731, Saudi Arabia; lukadel@yahoo.fr; 9 Faculty of Science of Sfax, Department of Chemistry, University of Sfax, B.P. 1171, 3000, Sfax 3029, Tunisia; 10 Laboratory of Genetic, Biodiversity and Valorization of Bioressources, Higher Institute of Biotechnology of Monastir, University of Monastir, Avenue Taher Hadded BP 74, Monastir 5000, Tunisia; 11 Department of Microbiology and Immunology, College of Medicine and Health Sciences, UAE University, Al Ain P.O. Box 17666, United Arab Emirates

**Keywords:** *Asphodelus tenuifolius*, LC-ESI/MS, phenolic compounds, antioxidants, antibacterial, antifungal, antiviral, ADME

## Abstract

*Asphodelus tenuifolius* Cav. (*A. tenuifolius*) is a medicinal plant with a long history of traditional use to treat ailments. In this study, total phenolic and flavonoid content evaluation using LC-ESI/MS analysis and various biological activities (antioxidant, antibacterial, antifungal, antiviral and cytotoxicity) of organic extracts from the aerial parts of *A. tenuifolius* were analyzed. ADME tools were used to predict the potential of the identified compounds from the most potent extract as specific drugs. As shown, LC-ESI/MS results of chloroformic extract allowed the tentative identification of 12 compounds. Chloroformic extract was rich in polyphenols and flavonoids and exhibited the highest antioxidant activity given by DPPH (IC_50_ = 25 µg/mL) as compared to the BHT standard (11.5 µg/mL) and β-carotene bleaching assays (IC_50_ = 95.692 µg/mL). Antibacterial activity results showed that chloroformic extract has a highest activity against Gram-positive and -negative bacteria, especially against *Salmonella* Typhimurium DT104 (IZ = 19.3 mm, MIC = 18.75 mg/mL, MBC = 37.5 mg/mL). The MBC/MIC ratio was evaluated to interpret the activity that was bacteriostatic rather than bactericidal. Conversely, weaker antifungal activity was registered, and no antiviral activity was observed for all extracts against Herpes Simplex Virus type 2 and Coxsakievirus B-3 viruses. Cytotoxic activity on VERO cell line results revealed that butanol extract was not toxic, with CC_50_ value of 1430 µg/mL, while chloroformic extract showed moderate cytotoxicity. Additionally, in silico studies performed proved promising pharmacokinetic and drug-likeness properties of the main compounds from the chloroformic extract. Taken together, this work highlights the potent bioactivity and acceptable drug-likeness of this plant, which supports its further preclinical development.

## 1. Introduction

The *Liliaceae* family is a rich source of natural products displaying a vast range of structural diversity. It includes approximately 289 genera and 4000 grown species, with many important medicinal, edible and ornamental plants [1]. Among these genera, eighteen species of *Asphodelus* have been widely cultivated throughout the Mediterranean area, North Africa and Southeast Asia. It reaches its maximum diversity in the Iberian Peninsula and Northwest Africa [2]. *Asphodelus* species were found to contain several secondary metabolites such as flavonoids [3,4], anthraquinones [5,6], phenolic acids [7,8], triterpenes [9,10], fatty acids [11] and naphthalene derivatives [12]. Different ethnomedical uses were ascribed to *Asphodelus* species, including *Asphodelus tenuifolius* Cav., known as onion weed [13], native to the Mediterranean region, which is used traditionally not only as a vegetable but also for colds, hemorrhoids, rheumatic pain, diuretic agent and wound healing [14]. As recently overviewed [15], organic extracts from the whole plant, fruits, roots, seeds and leaves were studied for samples collected from different harvest regions in the world [13,16,17,18,19,20,21] and also specifically from Algerian regions [14,22]. The diversity of results is mainly due to the harsh climatic conditions, which stimulate the biosynthesis of secondary metabolites, different plant growing conditions, collecting regions and different extraction procedures applied [23,24].

In the present study, we set out to provide more information on the *A. tenuifolius* phytochemical composition by LC-ESI/MS analysis. In addition, evaluation of total phenolic and flavonoids contents of chloroformic, ethyl acetate and butanol extracts was performed, investigating their antioxidant, antibacterial, antifungal, cytotoxic and antiviral activities. To the best of our knowledge, this work is the first report on chemical composition study based on RP-HPLC-ESI/MS measurements of *A. tenuifolius* plant extracts. Additionally, ADME/pharmacokinetics and drug-likeness properties of the identified metabolites were carried out through in silico SwissADME online program.

## 2. Materials and Methods

### 2.1. Chemical and Reagents

Folin-Ciocalteu reagent, sodium carbonate anhydrous (Na_2_CO_3_), gallic acid, sodium nitrite solution (NaNO_2_), sodium hydroxide (NaOH), sodium chloride (NaCl), aluminum chloride hexahydrate solution (AlCl_3_-6H_2_O), 2,2-Diphenyl-1 picrylhydrazyl (DPPH), catechin and β-carotene were from Fluka (Buchs, Switzerland). Linoleic acid and Tween 20 were purchased from Sigma-Aldrich (GmbH, Sternheim, Germany). Sulfuric acid (H_2_SO_4_) was obtained from Merck (Darmstadt, Germany). All analytical grade solvents were purchased from Sigma-Aldrich (Milan, Italy), with the exception of solvents for LC–MS analysis, which were HPLC–MS grade.

### 2.2. Plant Material and Extraction Procedure

The whole plant of *A. tenuifolius* Cav. was collected in May 2012 from southwest Algeria and identified by an expert botanist M. Mohamed Ben Abd-elhakem (Ex-Director of the National Agency of Preservation of Natural Resources, Bechar, Algeria). An authenticated voucher specimen with the identification number (AS10TEN) was deposited at the herbarium of the VARENBIOMOL research unit, University Mentouri Constantine. A total of 1250 g of the whole plant was dried, powdered and extracted with 80% ethanol aqueous solution at room temperature (each extraction lasting about 48 h). After filtration and concentration under vacuum at about 40 °C, the combined concentrated ethanol extract was suspended in distilled water. Each resulting solution was extracted successively using chloroform, ethyl acetate and butanol. The organic phases were filtered and concentrated in a vacuum at 38 °C to obtain dry extracts: chloroform (CHE, 3.60 g), ethyl acetate (EAE, 4.05 g) and butanol (BE, 6.40 g).

### 2.3. Total Phenolic Content (TPC)

Total phenolic content (TPC) was determined using Folin-Ciocalteu method according to Dewanto et al. [25] using Gallic acid as a standard. The reference range was prepared with Gallic acid in different concentrations from 50 to 500 µg/mL. Total phenolic contents are expressed as milligram Gallic acid equivalents per gram of dry residue (mg GAE/g DR). All samples were analyzed in three replicates.

### 2.4. Total Flavonoids Content (TFC)

Total flavonoids content (TFC) was measured using the colorimetric method introduced by Dewanto et al. [25] with few modifications. A calibration curve was constructed using catechin standard solution in different concentrations from 50 to 500 µg/mL. Total flavonoids contents were calculated as catechin equivalents per gram of plant dry residue (mg CE/g DR). Measurements were performed at least in triplicate.

### 2.5. LC-ESI/MS Analysis

LC-ESI/MS profiles were performed using a Hewlett–Packard (Palo Alto, CA, USA) Model 1100 Series liquid chromatography coupled to a Photo Diode Array detector (Agilent, Palo Alto, CA, USA) 1100 Series, and to an Esquire LC-ion trap mass spectrometer (Bruker Daltonics, Billerica, MA, USA) equipped with an electrospray ionization (ESI) interface. Separation was achieved on a Phenomenex Luna C18 analytical column (250 mm × 4.6 mm i.d.; 5 μm particle diameter, end-capped). The mobile phase consisted of water (eluent A) and acetonitrile (eluent B) at a flow rate of 1 mL/min. The injection volume was 2 µL. Gradient elution was carried out using the following timetable: 20% A and 80% B in 30 min, then 100% B in 40 min. The resulting total run time was 70 min. The Photo Diode Array detector was set at a range of 200–700 nm for all peaks. The chromatogram was recorded at 215 nm, 254 nm, 300 nm and 330 nm.

### 2.6. NMR Analysis

NMR experiments were performed on: (1) Bruker DRX-600 spectrometer (Bruker BioSpin, Rheinstetten, Germany) equipped with a Bruker 5 mm TCI CryoProbe at 300 K. (2) Bruker-Avance 400 spectrometer by using a 5 mm BBI probe. NMR spectra were acquired in CDCl_3_, DMSO-*d_6_*, CD_3_COCD_3_ and CD_3_OD in the phase-sensitive mode with the transmitter set at the solvent resonance and time-proportional phase increment (TPPI) used to achieve frequency discrimination in the ω_1_ dimension. The standard pulse sequence and phase cycling were used for HSQC and HMBC experiments.

### 2.7. Evaluation of Antioxidant Activity

#### DPPH Radical-Scavenging Activity and β-Carotene Bleaching Assay

The effect of the various tested extracts on DPPH-degradation was estimated according to the method described by Espín et al. [26] while β-carotene linoleic acid bleaching assay was done according to Condelli et al. [27].

DPPH scavenging effect (%) = [(A_0_ − A_1_) × 100]/A_0_; (1)

where A_0_ is the absorbance of the blank, and A_1_ is the absorbance of the sample.

Results were expressed as percentage of β-carotene bleaching inhibition (AA%) and calculated as follows (Equation (2)):

AA% = (A β-carotene after 180 min/A initial β-carotene) × 100;(2)

where A_0_ and A_1_ have the same meaning as in Equation (1). The results are expressed as IC_50_ values (µg/mL).

### 2.8. Antibacterial and Antifungal Activities

#### 2.8.1. Disk Diffusion Assay

The antibacterial and antifungal activities of *A. tenuifolius* organic extracts were evaluated by the agar disk diffusion method described by Rios and Recio [28] and Snoussi et al. [29]. Eight strains generally recognized as the most important pathogens affecting food dishes (*Escherichia coli* ATCC 35218, *Vibrio parahaemolyticus* ATCC 17802, *Staphylococcus aureus* ATCC 25923, *Salmonella typhimurium* DT 104, *Staphylococcus epidermidis* CIP 106510, *Salmonella typhimurium* ATCC 1408, *Bacillus cereus* ATCC 11778, *Listeria monocytogenes* ATCC 19115) were tested in the present study. On other hand, the antifungal activity effect was tested against four *Candida* strains (*C. tropicalis* 06-85, *C. parapsilosis* ATCC 22019, *C. krusei* ATCC 6258 and *C. albicans* ATCC 2019). The same technique was used to evaluate the antifungal activity. Ampicillin (10 mg/mL) and Amphotericin B (10 mg/mL) were used as a positive control [30]. The antibacterial activities were evaluated by measuring the diameter of the growth inhibition zone (IZ) around the discs using a flat rule. All tests were performed in triplicate, and the mean diameter of IZ was calculated. The results were expressed in terms of IZ of growth around each disc in millimeters, considered as low activity (IZ from 1 mm to 6 mm), moderate activity (7 mm to 10 mm), high activity (11 mm to 15 mm) and very high activity (16 mm to 20 mm) [31].

#### 2.8.2. Microdilution Assay: MICs and MBCs Determinations

Minimal inhibitory concentrations (MICs) and minimal bactericidal concentrations (MBCs) values were determined according to the method described by Gormez et al., Boulaaba et al. [32,33] and Snoussi et al. [29].

### 2.9. Cytotoxic and Antiviral Activities

The cytotoxic activity was evaluated according to the method described by Snoussi et al. [30] on VERO (African green monkey kidney) cells line. The 50% Cytotoxic Concentration (CC_50_), defined as the concentration of the extract able to reduce of 50% the cell viability, was determined by regression analysis in comparison to negative control. The extracts that demonstrated activity at or below 100 µg/mL were categorized as having strong cytotoxic activity. Consequently, CC_50_ values between 100 µg/mL and 500 µg/mL were categorized as having moderate cytotoxicity, the extracts that had CC_50_ values between 500 µg/mL and 1000 µg/mL were considered to have weak cytotoxic activity [34] and the extracts had CC_50_ values more than 1000 µg/mL were considered to be nontoxic [35]. Antiviral activity was also evaluated according to the method reported by Snoussi et al. [30] on two viruses, Herpes Simplex Virus type 2 (HSV-2) and Coxsakievirus B-3 (CVB-3).

### 2.10. In Silico ADME Profiles

The pharmacokinetics and drug-likeness properties of identified compounds from CHE of *A. tenuifolius* were estimated using ADME (absorption, distribution, metabolism and excretion) descriptors through SwissADME online server (http://www.swissadme.ch/ (accessed on May 2020)) by entering chemical structure followed by SMILES [36,37]. 

### 2.11. Statistical Analysis

The results were given as the average ± SD for three replicates. The IC_50_ of DPPH, the CC_50_ and the antiviral IC_50_ were calculated by linear regression analysis. The β-carotene bleaching method values, the total secondary metabolite contents and the inhibition zone determination were performed using Microsoft Excel. The data were subjected to Duncan’s multiple range tests. The statistical analyses were determined with the SPSS statistical software program (SPSS v.16), and *p* values < 0.05 were regarded as significant.

## 3. Results and Discussion

### 3.1. Phytochemical Contents

The chloroformic extract was rich in phenolic constituents (40.99 mg GAE/g DR) compared to the rest of the studied *A. tenuifolius* extracts (Table 1). This can be attributed to the higher solubility of constituents containing phenolic rings in this extract, whereas butanol extract was the one with the lowest phenolic concentration (10.54 mg GAE/g DR), suggesting their weaker solubility in this extract. In fact, Mahboub et al. [22] reported different results obtained from lyophilized samples of *A. tenuifolius* harvested from septentrional Algerian Sahara (101.82 µg GAE/g DR). In the same way, Munir et al. [38] and Al-Laith et al. [39] reported other data on *A. tenuifolius* from Pakistan and Bahrain, respectively, with total phenolic contents ranging within 53.40 to 76.23 mg GAE/g DR and 139.66 to 442.44 mg GAE/g DR, respectively, for various solvent extracts and harvested sites.

Results of total flavonoid contents are shown in Table 1. Chloroformic extract displayed also the highest flavonoid contents (213.07 mg CE/g DR), followed by EAE (202.89 mg CE/g DR). Previously, Mahboub et al. [22] found a good level of flavonoid contents marked in lyophilized dried extracts with a value of 16.10 µg QE/g DR. Similarly to our results, Munir and colleagues [38] also found flavonoid contents in the range of 165.82 to 312.12 mg QE/g DR for all studied extracts. The presence of phenolics and flavonoids is very important to assess the antioxidant potency, especially due to their chemical structure affecting redox properties, which play a vital role in absorbing and neutralizing free radicals [40,41].

In summary, the lowest polar solvent (CHE) displayed the highest phenolic and flavonoid contents, and the higher polar solvent (BE) showed the lowest amount, indicating the richness of *A. tenuifolius* extract in low polar phenolic compounds.

### 3.2. HPLC-DAD-ESI/MS Analysis

**Figure 1:**
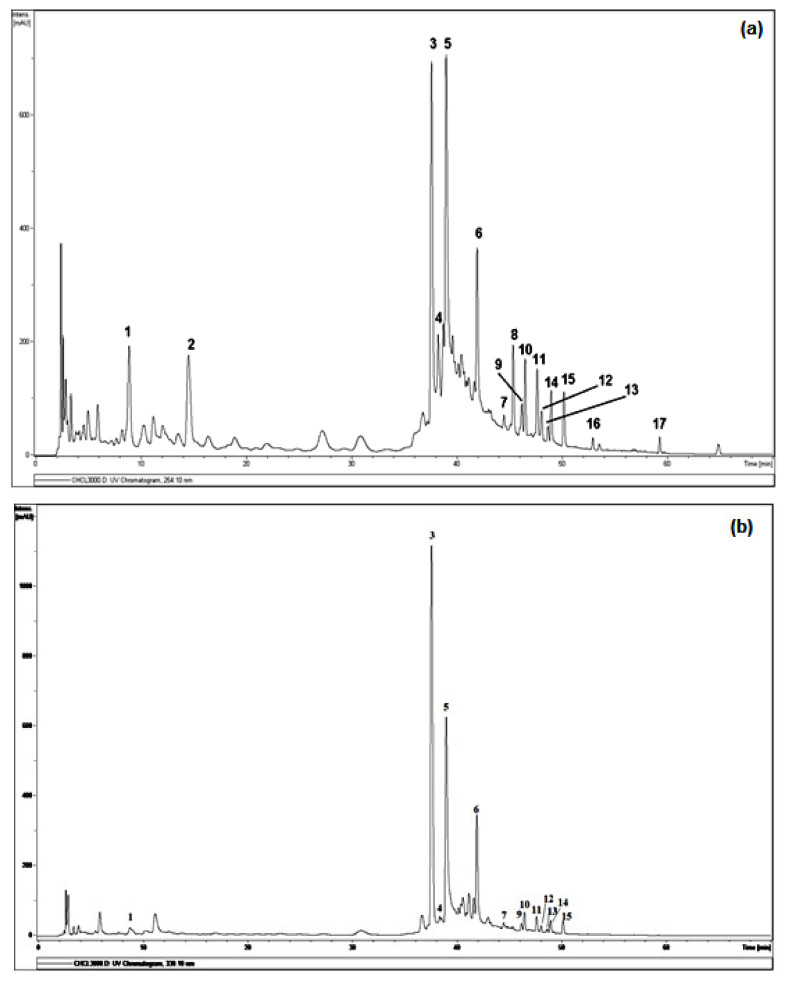
DAD-HPLC profile detected at 254 nm (**a**) and 330 nm (**b**) of LC–MS analysis on CHE. Legends: (**1**): Apigenin-7-*O*-glucoside, (**3**): Tamgermanetin, (**5**): Luteolin, (**6**): Apigenin, (**7**): Ramosin, (**8**): Aloe-emodin, (**10**): (*P*,10′*S*)-oxanthrone-10′-*β*-glucopyranosyl asphodelin, (**12**): (*M*,10′*S*)-oxanthrone-10′-*β*-glucopyranosyl asphodelin, (**13**): (10′*R*)-oxanthrone-10′-*β*-D-xylopyranoside asphodelin, (**14**): (10′*S*)-oxanthrone-10′-*β*-D-xylopyranoside asphodelin, (**15**): (10′*S*)-oxanthrone-10′-*β*-L-arabinopyranoside asphodelin, (**17**): Asphodeline. Peaks **2**, **4**, **9**, **11** and **16** are still unidentified.

A qualitative analysis of constituents present in chloroformic extract was performed by LC-ESI/MS. As shown in Figure 1, the full scan using negative electrospray ionization mode revealed the presence of several phenolic compounds, based on the over 17 major peaks detected within 70 min of elution. Table 2 summarizes retention time (Rt), *m/z*, molecular weight and formula of compounds identified or deduced based on data reported in the literature. All tentatively identified compounds are already known from *A. tenuifolius* plant and *Asphodelus* genus, while some minor metabolites are still unidentified.

**Figure 2:**
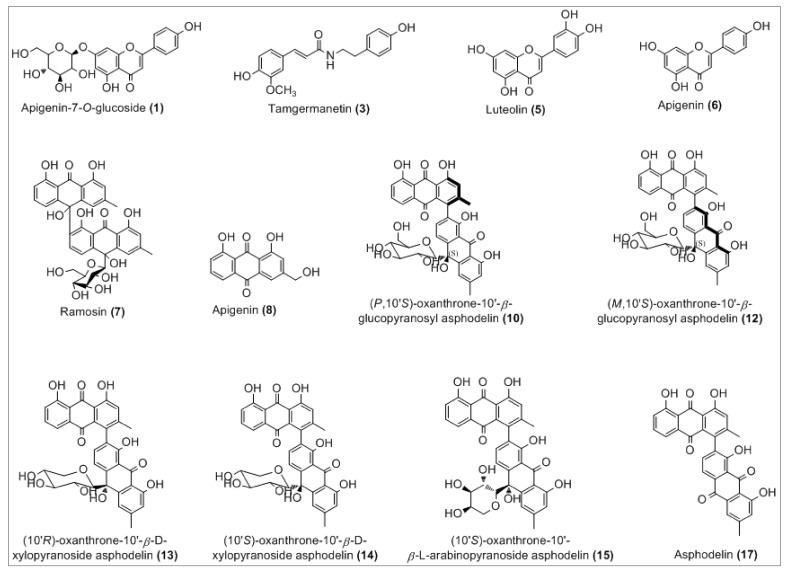
Chemical structures of the tentatively identified compounds.

The analysis of the chloroformic extract leads to the tentative identification (Figure 2) of 12 compounds of 17 detected by comparison with the respective reported literature data, both full and fragmentation ESI-MS ion peaks [M-H]^−^ and supported by 1D and 2D NMR analysis.

The complete list of the identified phytoconstituents is as follows: (**1**) apigenin-7-*O*-glucosyl (*m*/*z* 431.3), which was never reported in *A. tenuifolius* but reported previously from *Asphodelus ramosus* by Reynaud et al. [42]; (**3**) tamgermanetin (*m*/*z* 312.2) reported by Faidi et al. [17] from *A. tenuifolius*; (**5**) luteolin (*m*/*z* 284.9) reported in many plants of *Asphodelus* genus [4,8,17,43]; (**6**) apigenin (*m*/*z* 269.0) found in *A. tenuifolius* by Faidi et al. and Di Petrillo et al., from *Asphodelus microcarpus* [4,17]; (**7**) Ramosin (*m*/*z* 671.0) reported only one time by Adinolfi et al. from *A. ramosus* [44]; (**8**) Aloe-emodin (*m*/*z* 269.0) described by Hammouda et al. from *Asphodelus fistulosus* and van Oudtshoorn from *Asphodelus albus* [45,46]. Recently, Khalfaoui et al. [47] found both compounds (**10**) *P*,10′*S*-oxanthrone-(10′)-*β*-glucopyranosyl asphodelin (*m*/*z* 668.9) and (**12**) *M*,10′*S*-oxanthrone-(10′)-*β*-glucopyranosyl asphodelin (*m*/*z* 668.9) from *A*. *tenuifolius*. Compounds (**13**) 10′*R*-oxanthrone-(10′)-*β*-D-xylopyranoside asphodelin (*m*/*z* 638.9), (**14**) 10′*S*-oxanthrone-(10′)-*β*-D-xylopyranoside asphodelin (*m*/*z* 639.0) and (**15**) 10′*S*-oxanthrone-(10′)-*β*-L-arabinopyranoside asphodelin (*m*/*z* 639.0) reported previously by Ghoneim et al.from *A. microcarpus* [48]. Finally, (**17**) asphodelin (*m*/*z* 505.0) reported in many studies from *A. acaulis*, *A. albus*, *A. fistulosus* and *A. microcarpus* [12,43,45,49]. However, compounds corresponding to peaks **2**, **4**, **9**, **11** and **16** are still unidentified.

In detail, the Mass spectrum (MS) recorded in negative ion mode showed a molecular deprotonated ion at *m*/*z* 431.3, it fragmented giving as base peak the radical aglycone ion at *m*/*z* 269, corresponding to the loss of a glucose moiety and assigned to apigenin glycoside (**1**). For peak (**3**), the MS base peak at *m*/*z* 312.2 also showed two peaks (*m*/*z* 178.1 and 135.1) which are characteristic of the tamgermanetin. Compound eluted at peaks (**8**) showed a molecular deprotonated ion at *m*/*z* 269.2 corresponding to an Aloe-emodin with a characteristic fragment at *m*/*z* 240 [M-H-CHO]^−^. Results showed also molecular deprotonated ion at *m*/*z* 668.9 for four peaks (**9**, **10**, **11** and **12**) observed in different retention times with the same fragment at *m*/*z* 506.0 due to the loss of a hexoside moiety [M-H-162]^−^. Regarding Khalfaoui et al. [47], the peak (**10**) corresponds to (*P*,10′*S*)-oxanthrone-10′-*β*-glucopyranosyl asphodelin and (**12**) to (*M*,10′*S*)-oxanthrone-10′-*β*-glucopyranosyl asphodelin. However, both peaks **9** and **11** are still unidentified, and the exact structure cannot be assigned; only planar structure can be assigned on the basis of all available spectral data similarly to peaks **10** and **12** as (10′*S)*-oxanthrone-10′-*β*-glucopyranosyl asphodelin. The same value for the molecular deprotonated ion at *m*/*z* 639.0 was observed for peaks **13**, **14** and **15** with the common fragment at *m*/*z* 506.1 due to the loss of xylose unit corresponding to (10′*R*)-oxanthrone-10′-*β*-D-xylopyranoside asphodelin (**13**), (10′*S*)-oxanthrone-10′-*β*-D-xylopyranoside asphodelin (**14**) and loss of arabinose unit corresponding to (10′*S*)-oxanthrone-10′-*β*-L-arabinopyranoside asphodelin (**15**) [48].

Although many phytochemical studies were previously reported on *A. tenuifolius* collected from many regions [13,14,16,17,18,19,20,21,22], but no LC-ESI/MS analysis was performed, and it is reported here in this study for the first time.

### 3.3. Antioxidant Activities

Antioxidant activity of different Algerian *A*. *tenuifolius* extracts was evaluated using two different tests: DDPH radical scavenging activity and β-carotene bleaching. The results presented in Table 1 were expressed as IC_50_ values and compared to the positive control BHT. As shown, all extracts displayed potent free radical scavenging activity on DPPH with IC_50_ values ranging from 25 to 92 µg/mL, clearly less important than thatpositive control BHT (11.5 ± 0.01 µg/mL). CHE had the strongest DPPH radical scavenging activity (25 ± 4.36 µg/mL) followed by EAE (45 ± 2.88 µg/mL), and BE (92 ± 4.05 µg/mL). In our results, the extract rich in flavonoids and phenolics (CHE) was found to be the most significant scavenger of DPPH radical, which is supported by the good correlation between phenolic and flavonoid contents and DPPH outcomes. Polyphenolic compounds are usually the major antioxidants in plant extracts [50]. Based on LC-ESI/MS results, chloroformic extract showed an important richness, especially in apigenin-7-*O*-glucoside, apigenin, luteolin, anthraquinones and their derivatives formerly known for their antioxidant roles [51,52,53,54,55].

Previous studies on methanol, ethanol and petroleum ether extracts of Algerian *A. tenuifolius* displayed an important antioxidant activity, with IC_50_ values ranging between 28.34 and 75.91 µg/mL [14]. Similarly, Al-Laith et al. [39] reported that, for extracts of the plant collected from two different harvested sites from Bahrain, IC_50_ values of antioxidant and antiradical activity varied between 18.37 and 37.24 mg/mL. Kalim et al. [18], also reported DDPH radical scavenging results from Indian *A*. *tenuifolius* with an IC_50_ = 2.00 µg/mL of 50% methanolic extract. This difference could also be due to the variable plant growing conditions, collection regions and extraction procedures [56,57].

Table 1 depicts the inhibition of β-carotene bleaching by *A. tenuifolius* extracts. It is possible to note that EAE had a significant activity, with an IC_50_ value of 73.581 µg/mL, close to BHTreported a study on a whole Indian *A. tenuifolius* methanol extract against two Gram-positive bacteria (*S. aureus* and *B. cereus*), with IZ diameters of 9 and 13 mm, respectively, while acetone extract showed only activity against a Gram-negative bacteria *K. pneumoniae* (IZ = 17 mm). Additionally, Panghal et al. [20] outlined an antibacterial study of Indian *A. tenuifolius* fruits using six organic solvents for extraction, in which no activities were observed for petroleum ether and aqueous extracts against all tested bacterial strains, specifically against *E*. *coli* for the aqueous extract (IZ = 13.67 ± 0.5). The same study revealed that benzene extract exhibited very good susceptibility to *K. pneumoniae*, *P. aeruginosa* (IZ = 10.33 ± 0.5) and *A. fumigatus* (IZ = 10.66 ± 0.5). In the same way, all other extracts displayed antibacterial activity against overall tested bacterial strains. Moreover, a good antibacterial activity was observed by Dangi et al. and Menghani et al. [16,19] against the number of selected bacteria. From Indian *A. tenuifolius*, various extracts were found to be active against almost all the tested bacteria. Eddine et al. and Mahboub et al. [14,22] reported studies from Algerian *A. tenuifolius*, collected from the south-east and septentrional Sahara of Algeria, respectively. In detail, the results on the antibacterial effect of methanol, ethanol and petroleum ether extracts reported by Eddine et al. [14] showed a marked activity against *S. aureus* and *P. putida* (IZ = 11 mm), *B. cereus*, *P. aerigunosa*, *A. tumefaciens* and *E. coli* (IZ = 10 mm), and *S. Arizona* (IZ = 9 mm). Mahboub et al. [22] found a remarkable inhibition against *E. coli* and *P. aeruginosa* for the lyophilized sample, unlike our results on chloroformic, ethyl acetate and butanol extracts.

Table 3 shows that antibacterial effect of the extracts was more important than the antifungal one, suggesting that yeast strains are more resistant to bacteria. These results are consistent with the ones previously reported, indicating that the inhibitory activity is pathogen-specific and depends on a number of factors, including the solvents, concentration of the crude drug, temperature, plant parts used for the extraction of secondary metabolites and rate of diffusion [58]. Both MICs and MBCs of *A*. *tenuifolius* organic extracts are summarized in Table 3. MICs ranged from 0.58 to 37.5 mg/mL, from 0.39 to 25 mg/mL, from 0.78 to 50 mg/mL for CHE, EAE and BE, respectively, for all bacterial and fungal strains tested. Regarding MBCs, high concentrations were needed to eliminate the growth of all tested bacterial and fungal strains, with values ranging from 2.34 to >100 mg/mL. Results indicated that CHE was the most effective compared to data obtained for EAE and BE. Our values of MICs and MBCs parameters are different from those obtained in numerous studies, specifically by Soliman et al. [59], in which MICs ranged from 25 to 50 µg/mL; Faidi et al. [17], found values from 0.15 to 4.1 mg/mL; Dangi et al. [16], with MICs results in range from 8 to 32 µg/mL; and Panghal et al. [20], reporting values in the range from 31 to 500 µg/mL. This behavior could be related mainly to the difference in the phenolic composition of each studied extract, plant origin, tested microorganisms and the size of the inoculum [60]. 

The inhibitory effect of *A. tenuifolius* extracts was evaluated against four yeasts. The results revealed weaker antifungal potency as compared with the standard, and Amphotericin B. EAE seems to be the most effective, especially against *Candida albicans*, followed by BE and CHE. In fact, *C. albicans* ATCC 2019 was found to be the most sensitive to *A. tenuifolius* extracts, while *C. krusei* ATCC 6258 was the most resistant yeast. In addition, the MIC values related to the extraction solvents on the four *Candida* species are similar (ranging from 12.5 to 37.5 mg/mL). Moreover, concentrations ranging from 50 to >75 mg/mL for almost all tested *Candida* strains were sufficient to reproduce a fungicidal effect. Amphotericin B was more efficient on the four tested *Candida* species in comparison with the three types of extract tested with low MIC values, ranging from 0.024 to 0.195 mg/mL, and low MFC values (0.39 to 6.25 mg/mL). Previous results have shown a moderate antifungal activity of *A. ramosus* L., and *A. tenuifolius* L. tested on *C. albicans*, *C. dubliniensis*, *C. glabrata* and *C. krusei,* with a diameter of growth inhibition zone ranging between 10 and 16 mm [61]. Recently, Soliman et al. [59] reported that the ethanolic extract of *A. tenuifolius* (100 μg/mL) inhibits the growth of *C. albicans* on Lauria Bertani agar with GIZ = 16 ± 0.5 mm. Additionally, Salhi et al. [62] reported that the *A. tenuifolius* aqueous extract (at 20%) was able to inhibit mycelial growth in *Fusarium graminearum* with a percentage of about (60.34%). 

According to reported data [33,63], an extract has bacteriostatic effect when the ratio MBC/MIC is more than 4 and a bactericidal effect if the ratio MBC/MIC is less than 4. In our study, and based on the obtained results, the effect of CHE was bacteriostatic rather than bactericidal. 

### 3.4. Cytotoxic and Antiviral Activities

Our results showed that only BE was safe and non-toxic, with CC_50_ value of about 1340 µg/mL, while chloroformic extract and ethyl acetate extract exhibited moderate cytotoxicity on VERO cell line, with CC_50_ values of 400 and 333 µg/mL, respectively, indicating the presence of some cytotoxic compounds responsible for the observed toxicological activity [47]. Consistent with our results, Soliman et al. [59], testing different *A*. *tenuifolius* extracts at different concentrations by using fresh human erythrocytes, reported that all extracts are safe and not toxic. 

The antiviral activity was evaluated for the first time on *A*. *tenuifolius* extracts against Herpes Simplex Virus type 2 (HSV-2) and Coxsackievirus B-3 (CVB-3), but results showed that no extracts were active, despite the high number of polyphenols reported in the literature to possess inhibitory activity against viruses [64].

### 3.5. ADME Predictions

Pharmacokinetic and drug-likeness properties were evaluated using the SwissADME online program to predict whether the identified bioactive molecules become a starting scaffold or lead compounds toward future synthetic drug discovery. As shown (Table 4), out of the present compounds, only **3**–**6** and **8** were estimated to have high absorption in the gastrointestinal tract, which makes them a successful drug. Another advantage is that they are not P-gp substrate (except 1), which makes them a good candidate against multidrug-resistant cancer cells, overexpressing this drug transporter. They were predicted to not be blood–brain-barrier (BBB)-permeant, meaning that they are not able to cross the blood–brain barrier into the brain, where it binds to specific receptors. The prediction of putative drug–drug interaction through Cytochromes P450 (CYPs) inhibition, which affects the metabolism of numerous xenobiotics, demonstrates that only compounds **5**, **6** and **7** were expected to be inhibitors of CYP1A2. The compound **17** affected CYP2C19; those **7** and **10**, **12**–**15** and **17** affected CYP2C9; and **3**, **5** and **6** affected CYP2D6. These results suggest that all compounds may be metabolized by more than one enzyme, which in turn can minimize the risk of drug–drug interaction. In addition, eight of the twelve compounds were not inhibitors of the isoenzyme CYP3A4, which is largely implicated in the metabolism and elimination of the majority of clinically used drugs. As for the skin sensitivity prediction given by their logKp values, all tested compounds displayed negative values ranging from −8.95 to −5.24 cm/s, meaning that they are not accessible through the skin. Regarding their drug-likeness properties, more than half of the compounds considered were expected to have a good bioavailability score (0.55) and obeyed Lipinski’s rule of five.

**Figure 3:**
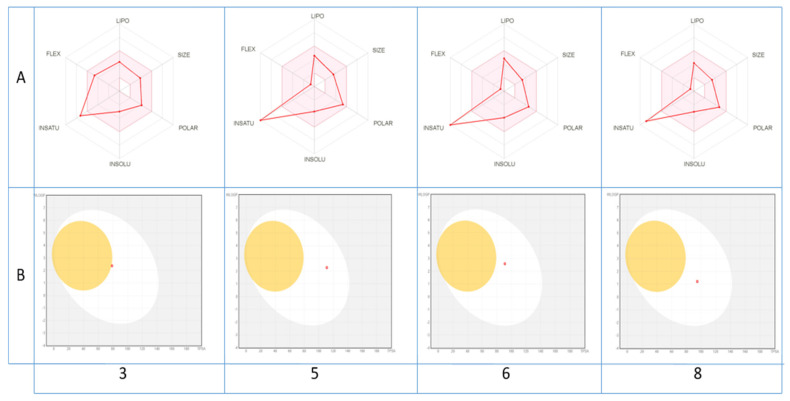
Bioavailability radar of (**A**) the top drug like phytoconstituents based on their suitable physicochemical indices ideal for oral bioavailability and boiled-egg model, (**B**) top bioavailable phytoconstituents using Swiss ADME predictor. LIPO, Lipophilicity: −0.7 < XLOGP3 < þ5; SIZE, Molecular size: 150 g/mol < mol. wt. < 500 g/mol; POLAR, Polarity: 20 Å2 < TPSA < 130 Å2; INSOLU, Insolubility: 0 < Log S (ESOL) < 6; INSATU, Instauration: 0.25 < Fraction Csp3 < 1; FLEX, Flexibility: 0 < Number of rotatable bonds < 9. The colored zone is the suitable physicochemical space for oral bioavailability.

The druglikeness of the identified molecules can be estimated also through a visualization of their bioavailability radar (Figure 3A), with the pink area representing the optimal range for each property (lipophilicity, size, polarity, solubility, unsaturation and flexibility).

As shown, all compounds fall entirely in the pink area (except for unsaturation fraction), suggesting their better drug-like properties. In contrast, their pharmacokinetic properties may be also predicted via the passive gastrointestinal absorption (HIA) and brain penetration (BBB) of the top bioavailable compounds as a function of the position of the molecules in the WLOGP-versus-TPSA referential. The depicted results of the BOILED-Egg model (Figure 3B) indicate clearly that only compounds **5**, **6**, **8** and to lesser degree **3** with a red point in the white ellipse have a high probability of being passively absorbed by the gastrointestinal tract and are non-substrate of the P-gp.

## 4. Conclusions

The aerial parts of Algerian *A. tenuifolius* were subjected to successive solvent fractionation, and chloroform extract was targeted. To the best of our knowledge, this work represents the first attempt to study the chemical composition by LC-ESI/MS analysis which led to the tentative identification of 12 compounds out of 17 detected, as well to study the biological activities, especially antibacterial, cytotoxic and antiviral, of the various solvent extracts. In this context, *A. tenuifolius* chloroformic extract gave interesting results in terms of both antioxidant and antibacterial activities. Our findings confirm the interesting potential of this plant as a valuable source of natural bioactive molecules that can be used in the food industry. The ADME of some isolated compounds from chloroformic extract demonstrate their good bioavailability and drug-likeness properties, especially tamgermanetin, luteolin, apigenin and aloe-emodin, which suggest, in parallel with in vivo and preclinical assays, a future new drug candidate.

## Figures and Tables

**Table 1:** Total phenolic and flavonoid contents expressed as gallic acid and catechin equivalents, in mg/g dry sample, respectively, DPPH radical-scavenging activity and β-carotene bleaching capacity.

Extracts	Total Phenolic Content (mg GAE/g DR)	Total Flavonoids Content (mg CE/g DR)	DPPH IC_50_ (µg/mL)	β-Carotene IC_50_ (µg/mL)
CHE	40.99 ± 0.41 ^c^	213.07 ± 1.72 ^c^	25 ± 4.36 ^a^	95.692 ± 0.027 ^b^
EAE	24.04 ± 0.55 ^b^	202.89 ± 6.15 ^b^	45 ± 2.88 ^b^	73.581 ± 0.087 ^a^
BE	10.54 ± 0.20 ^a^	62.85 ± 1.33 ^a^	92 ± 4.05 ^c^	97.775 ± 0.007 ^b^
BHT	-	-	11.5 ± 0.01	75 ± 0.2

Data were presented as mean of triplicate determinations ± standard deviation (*n* = 3). The letters (a–c) indicate a significant difference between the different antioxidant methods according to the Duncan test (*p* < 0.05).

**Table 2:** Phenolic profile by LC-ESI/MS analysis in negative ion mode of compounds **1**–**17** from CHE. Rt, retention time of the peaks detected in the chromatogram reported in Figure 1. NI = not identified.

Peaks	Rt (min)	MW	*m/z* [M-H]^−^	Molecular Formula	λ _max_ (nm)	Probable Compounds
1	8.9	432	431.3	C_21_H_20_O_10_	254, 330	Apigenin-7-*O*-glucoside
2	14.5	207	206.1	NI	254	NI
3	37.6	313	312.2	C_18_H_19_O_4_N	254, 330	Tamgermanetin
4	38.2	584	583.0	NI	254, 330	NI
5	39.0	286	284.9	C_15_H_10_O_6_	254, 330	Luteolin
6	41.6	270	269.0	C_15_H_10_O_5_	254, 330	Apigenin
7	44.5	672	671.0	C_36_H_32_O_13_	254, 330	Ramosin
8	45.3	270	269.2	C_15_H_10_O_5_	254	Aloe-emodin
9	46.1	670	668.9	C_36_H_30_O_13_	254, 330	NI
10	46.5	670	668.9	C_36_H_30_O_13_	254, 330	(*P*,10′*S*)-oxanthrone-10′-*β*-glucopyranosyl asphodelin
11	47.6	670	668.9	C_36_H_30_O_13_	254, 330	NI
12	48.0	670	668.9	C_36_H_30_O_13_	254, 330	(*M*,10′*S*)-oxanthrone-10′-*β*-glucopyranosyl asphodelin
13	48.6	640	639.0	C_35_H_28_O_12_	254, 330	(10′*R*)-oxanthrone-10′-*β*-D-xylopyranoside asphodelin
14	48.9	640	639.1	C_35_H_28_O_12_	254, 330	(10′*S*)-oxanthrone-10′-*β*-D-xylopyranoside asphodelin
15	50.1	640	638.8	C_35_H_28_O_12_	254, 330	(10′*S*)-oxanthrone-10′-*β*-L-arabinopyranoside asphodelin
16	52.9	594	593.3	NI	254	NI
17	59.2	506	505.0	C_30_H_18_O_8_	254	Asphodelin

**Table 3:** Antibacterial and antifungal activities (expressed as diameter of IZ ± SD, on mm), MIC and MBC values (mg/mL) of *A. tenuifolius* organic extracts.

**Tested Microorganisms**	**Chloroformic Extract**			**Ethyl Acetate Extract**			**Butanol Extract**			**Ampicillin (10 mg/mL)**		
	**GIZ ± SD**	**MIC**	**MBC**	**GIZ ± SD**	**MIC**	**MBC**	**GIZ ± SD**	**MIC**	**MBC**	**GIZ ± SD**	**MIC**	**MBC**
*Staphylococcus epidermidis* CIP 106510	13 ± 2 ^b^	0.58	2.34	6 ± 0 ^a^	25	>50	6 ± 0 ^a^	50	>50	21.33 ± 0.57 ^c^	0.078	0.625
*Staphylococcus aureus* ATCC 25923	9.67 ± 0.58 ^b^	2.34	9.37	9.67 ± 0.58 ^b^	3.12	6.25	6 ± 0 ^a^	25	>50	26.66 ± 0.57 ^c^	0.078	0.625
*Vibrio parahaemolyticus* ATCC 17802	8.67 ± 1.15 ^b^	18.75	75	6 ± 0 ^a^	25	>50	6 ± 0 ^a^	50	>50	13.33 ± 0.57 ^c^	0.011	3
*Listeria monocytogenes* ATCC 19115	6 ± 0 ^a^	37.5	>75	6 ± 0 ^a^	0.39	1.56	6 ± 0 ^a^	0.78	3.125	12.33 ± 0.57 ^b^	0.023	0.093
*Bacillus cereus* ATCC 11778	6 ± 0 ^a^	4.68	18.75	6 ± 0 ^a^	25	>50	6 ± 0 ^a^	50	>100	26 ± 1 ^b^	0.078	0.625
*Salmonella typhimurium* DT 104	19.3 ± 1.15 ^c^	18.75	37.5	10.7 ± 2.08 ^b^	3.12	6.25	6 ± 0 ^a^	50	>50	*	*	*
*Salmonella typhimurium* ATCC 1408	6 ± 0 ^a^	18.75	75	6 ± 0 ^a^	12.5	>25	6 ± 0 ^a^	50	>50	18 ± 1 ^b^	0.023	0.093
*Escherichia coli* ATCC 35218	6 ± 0 ^a^	9.37	37.5	6 ± 0 ^a^	25	>50	6 ± 0 ^a^	50	>50	12.33 ± 0.57 ^b^	0.023	0.093
**Tested Microorganisms**	**Chloroform Extract**			**Ethyl Acetate Extract**			**Butanol Extract**			**Amphotricin B (10 mg/mL)**		
	**GIZ ± SD**	**MIC**	**MFC**	**GIZ ± SD**	**MIC**	**MFC**	**GIZ ± SD**	**MIC**	**MFC**	**GIZ ± SD**	**MIC**	**MFC**
*Candida krusei* ATCC 6258	6 ± 0 ^a^	37.5	>75	6 ± 0 ^a^	25	>50	6 ± 0 ^a^	25	>50	12 ± 0 ^b^	0.097	0.195
*Candida albicans* ATCC 2019	6 ± 0 ^a^	18.75	75	6 ± 0 ^a^	12.5	25	6 ± 0 ^a^	25	>50	12.66 ± 0.57 ^b^	0.024	0.781
*Candida parapsilosis* ATCC 22019	6 ± 0 ^a^	37.5	>75	6 ± 0 ^a^	25	>50	6 ± 0 ^a^	25	>50	10.33 ± 0.57 ^b^	0.195	0.39
*Candida tropicalis* 06-085	6 ± 0 ^a^	37.5	>75	6 ± 0 ^a^	25	>50	6 ± 0 ^a^	25	>50	12 ± 0 ^b^	0.39	6.25

Inhibition Zone. Data are presented as mean of triplicate determinations ± standard deviation (*n* = 3). MIC: Minimal Inhibitory Concentration. MBC: Minimal Bactericidal Concentration. MFC: Minimal Fungicidal Concentration. The letters (a–c) indicate a significant difference between the different extracts inhibition zone according to the Duncan test (* p < 0.05). *: Not tested.

**Table 4:** Physicochemical properties, pharmacokinetics and drug-likeness of identified compounds according to SwissADME software.

Entry	1	3	5	6	7	8	10	12	13	14	15	17
GI absorption	Low	High	High	High	Low	High	Low	Low	Low	Low	Low	Low
BBB permeant	No	No	No	No	No	No	No	No	No	No	No	No
P-gp substrate	Yes	No	No	No	No	No	No	No	No	No	No	No
CYP1A2 inhibitor	No	No	Yes	Yes	No	Yes	No	No	No	No	No	No
CYP2C19 inhibitor	No	No	No	No	No	No	No	No	No	No	No	Yes
CYP2C9 inhibitor	No	No	No	No	Yes	No	Yes	Yes	Yes	Yes	Yes	Yes
CYP2D6 inhibitor	No	Yes	Yes	Yes	No	No	No	No	No	No	No	No
CYP3A4 inhibitor	No	Yes	Yes	Yes	No	Yes	No	No	No	No	No	No
Log Kp (cm/s)	−7.65	−6.72	−6.25	−5.80	−8.95	−6.66	−8.39	−8.39	−8.16	−8.16	−8.16	−5.24
Lipinski’s Rule	Yes	Yes	Yes	Yes	Yes	Yes	No	No	No	No	No	Yes
Bioavailability Score	0.55	0.55	0.55	0.55	0.55	0.55	0.17	0.17	0.17	0.17	0.17	0.55

## Data Availability

All data generated or analyzed during this study are included in this article.

## Abbreviations

ADME	Adsorption: distribution, metabolism and extraction
BBB	Blood–brain barrier
BE	Butanol extract
BHT	Butylated hydroxytoluene
CC	Cytotoxic conentration
CE	Catechin equivalent
CHE	Chloroform extract
CVB-3	Coxsackievirus B-3
CYP	Cytochrome P450
DR	Dry residue
EAE	Ethyl acetate extract
GAE	Gallic acid equivalent
GI	Gastrointestinal
HIA	Human intestinal adsorption
HSV-2	Herpes simplex type 2
IZ	Inhibition zone
MIC	Minimal inhibitory concentration
MBC	Minimal bactericidal concentration
NI	Not identified
NMR	Nuclear magnetic resonance
P-gp	P glycoprotein
TPSA	Total prostate specific antigen
